# A Rare Case of Semaglutide-Associated Small Bowel Obstruction Complicated by Acute Kidney Injury Requiring Dialysis

**DOI:** 10.7759/cureus.104376

**Published:** 2026-02-27

**Authors:** Suhasini Rallabandi, Mansi Sharma, Krishnamraju Kosuru, Rahul Kashyap

**Affiliations:** 1 Medicine, Banner Baywood Medical Center, Mesa, USA; 2 Internal Medicine, University of North Carolina Health Wayne, Goldsboro, USA; 3 Pediatrics, Banner Health Center, Gilbert, USA; 4 Medicine, Drexel University College of Medicine, Philadelphia, USA; 5 Research, Global Remote Research Scholars Program, Princeton Junction, USA; 6 Critical Care Medicine, Mayo Clinic, Rochester, USA; 7 Research, WellSpan Health, York, USA

**Keywords:** acute kidney injury, glp-1 receptor agonists, intestinal ileus, semaglutide, small bowel obstruction

## Abstract

Glucagon-like peptide-1 (GLP-1) receptor agonists are widely used in the treatment of diabetes mellitus and obesity. They have proven to be cardioprotective, neuroprotective, and renoprotective, and are now also being considered for drug addiction. There has been increasing demand for these medications over the last several years, with the most common side effects being nausea, vomiting, and constipation. Nausea and vomiting are known to improve over a period of time due to tachyphylaxis. We present the case of a 59-year-old Hispanic obese female on semaglutide for weight loss. Her medication dose was recently increased from 0.25 mg to 0. 5mg under her physician’s supervision. She presented with nausea, vomiting, and abdominal pain, which was later diagnosed as small bowel obstruction (SBO) from severe ileus, and developed acute kidney injury (AKI) requiring hemodialysis for several weeks. This case was also complicated because of the past medical history of autoimmune disorder and granulomatosis polyangiitis, which required additional testing to rule out a possible autoimmune etiology of the current presentation. To our knowledge, this is a rare case of semaglutide-associated SBO and concomitant AKI requiring temporary dialysis, highlighting the adverse effects of GLP-1 receptor agonists and the need for further studies to help formulate best practice guidelines.

## Introduction

Glucagon-like peptide-1 (GLP-1) receptor agonists, such as semaglutide, tirzepatide, and liraglutide, are widely used in the management of diabetes mellitus and for weight loss [1]. These medications are being called “miracle” [2] or “wonder” [3] drugs for their numerous benefits for the heart, liver, kidneys, and mental health. Their main mechanism of action is by stimulating insulin secretion, delaying gastric emptying, and acting on the central nervous system in satiety control. Consequently, from delayed gastric emptying and stimulation of neural circuitry, nausea (5-10%), diarrhea (10-15%), vomiting (15-30%), and constipation are commonly noted gastrointestinal (GI) adverse effects [4,5]. We report a rare case of functional bowel obstruction following semaglutide dose escalation, mimicking mechanical small bowel obstruction (SBO) and complicated by acute kidney injury (AKI) requiring dialysis. The association between GLP-1 receptor agonists and SBO remains poorly defined in the current literature. Existing data are primarily derived from observational studies, and a definitive consensus on causality has not yet been established [6].

## Case presentation

A 59-year-old Hispanic female with a past medical history of obesity with a BMI of 43 kg/m², diverticulitis, gastric ulcer, granulomatosis with polyangiitis, anxiety, and depression was admitted to the hospital due to concern for SBO. She reported nausea, vomiting, and abdominal pain for a three-day duration. She was seen in the emergency room, a day before this admission with similar symptoms for which she was sent home on oral antibiotics for suspected colitis. However, the patient returned the next day with persistent symptoms. She was unable to tolerate oral intake and was subsequently admitted to our floor.

Her medication history was significant for semaglutide, which she reported at 0.25 mg subcutaneous injections weekly for four weeks. The dose was increased to 0.5 mg weekly three days before this admission. She was also on oral azathioprine twice daily and rituximab infusion every four months for the last six years as immunosuppressive therapy for her granulomatosis polyangiitis. Her last colonoscopy was a few months ago, which was significant for internal hemorrhoids and sigmoid diverticulosis. Her past surgical history was significant for a cholecystectomy.

**Figure 1:**
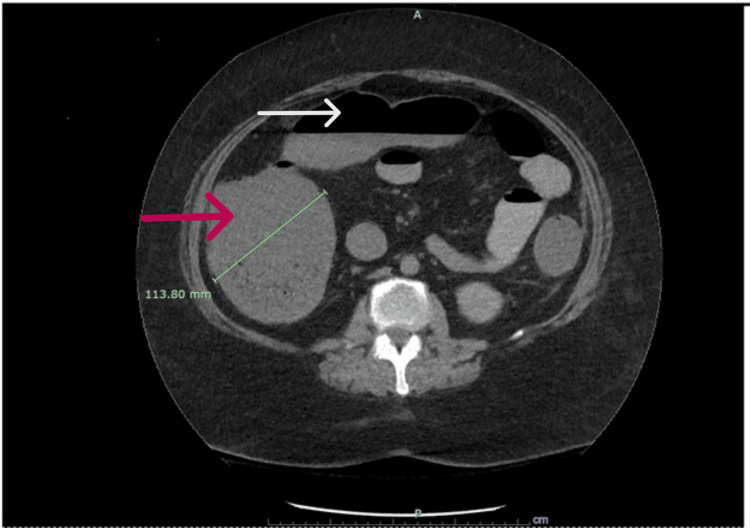
Cross-sectional image of CT of the abdomen without contrast. The white arrow is pointing toward the air fluid levels in the colon, suggesting a high small bowel obstruction. On the left side, the red arrow is pointing toward a severely distended colon measuring 11.3 cm, filled with stool.

Her physical examination showed mild abdominal distension and generalized abdominal tenderness with no rebound tenderness or guarding. Her vital signs were significant for hypotension with a blood pressure of 77/55 mmHg and tachycardia with a heart rate of 107 beats per minute. CT of the abdomen and pelvis showed moderately dilated small bowel loops and large bowel, which could be due to Ileus or early SBO, as can be seen in Figure 1.

**Figure 2:**
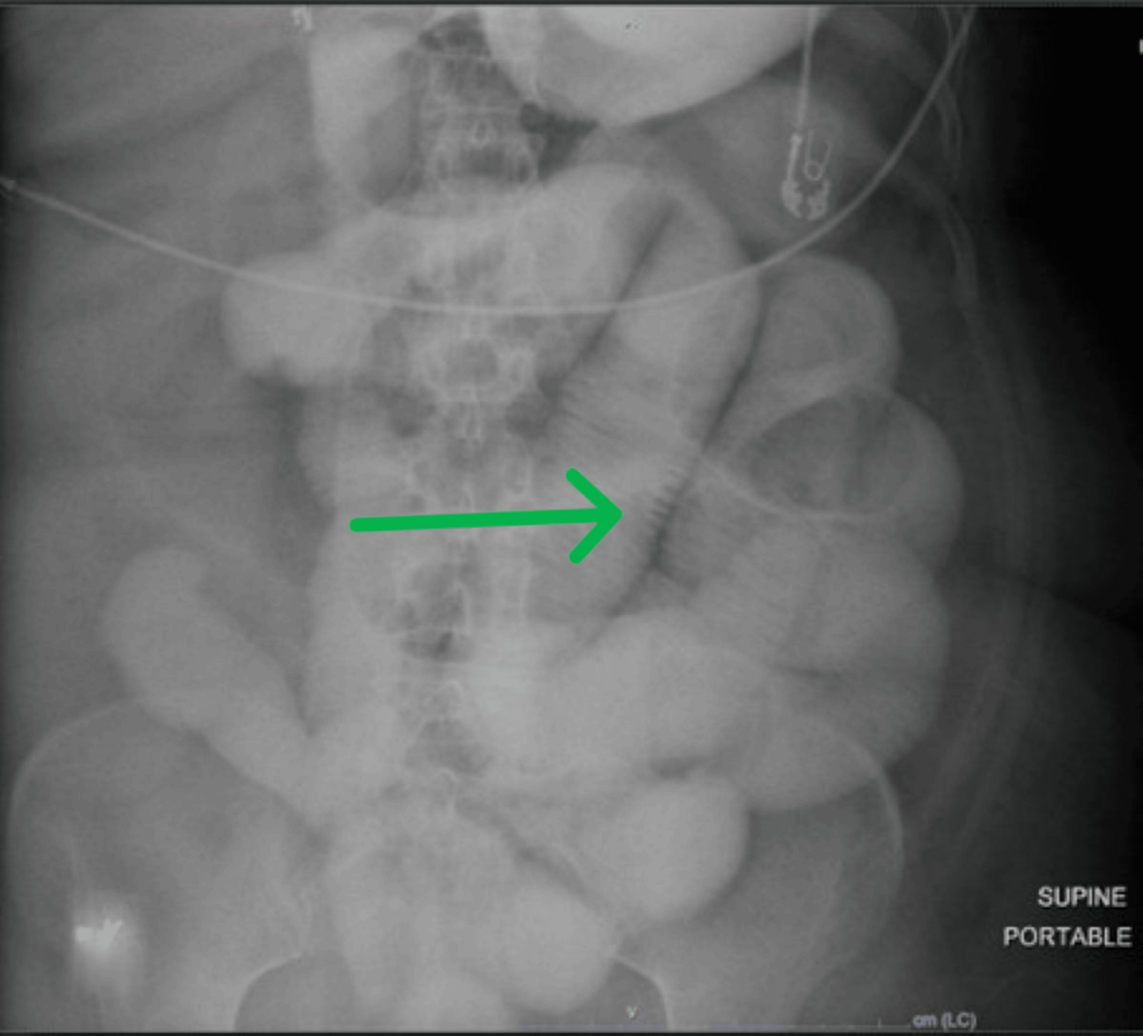
X-ray of the small bowel follow-through 24 hours after oral gastrografin study demonstrating persistent contrast in dilated small bowels, with the arrow pointing at valvulae conniventes, classic for small bowel distension, indicating high-grade small bowel obstruction.

**Figure 3:**
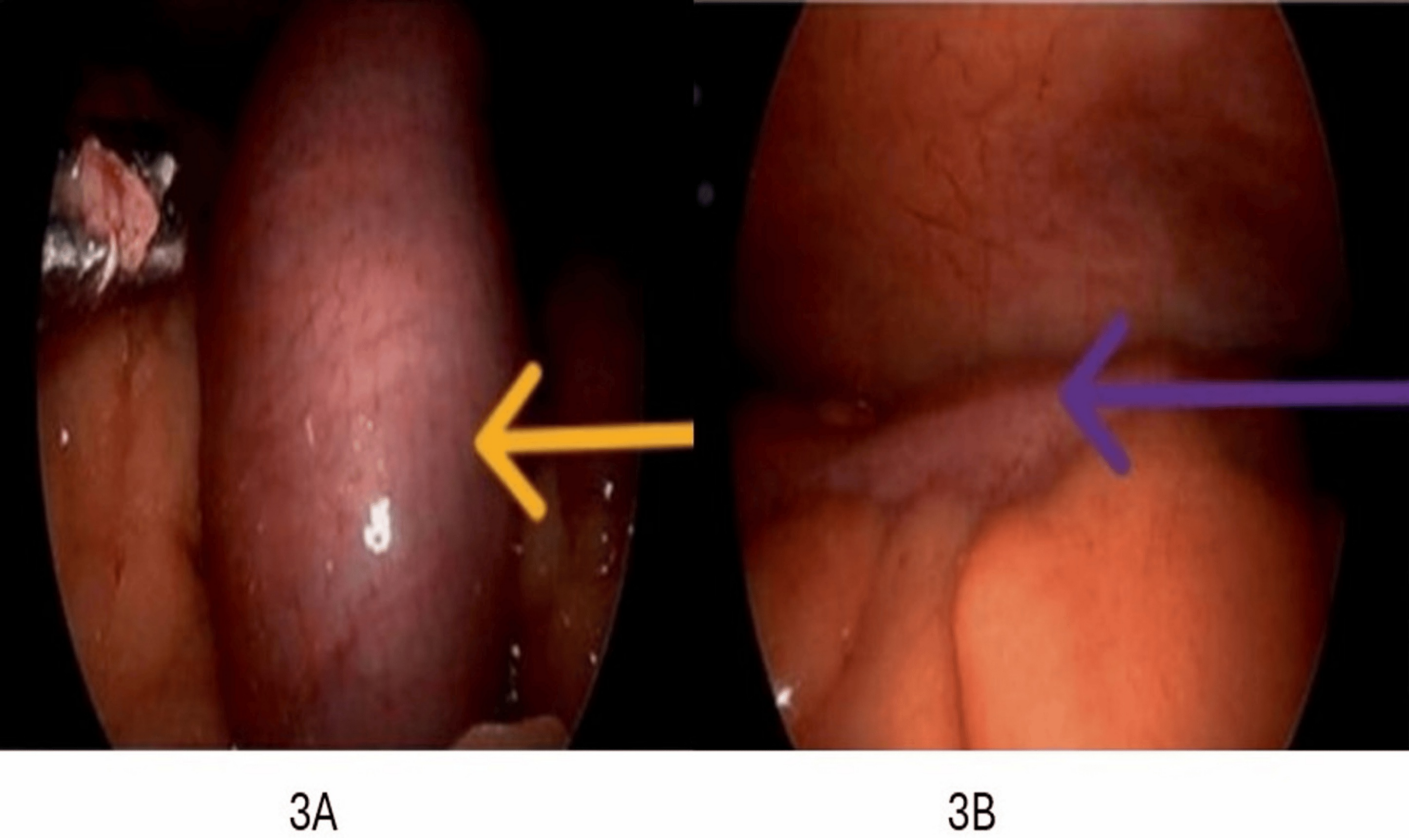
Surgical images from diagnostic laparoscopy. (A) The orange arrow is pointing toward the distended small bowel, suggestive of severe Ileus, ruling out mechanical bowel obstruction. (B) The purple arrow is pointing toward the intra-abdominal cavity with no signs of peritonitis.

The patient was admitted to the intensive care unit due to hypotension. She received 5 L of normal saline bolus with improvement in blood pressure. The X-ray small bowel follow-through, as can be seen in Figure 2, demonstrated high-grade SBO. Given the persistent findings of bowel obstruction on imaging, there was a high suspicion of mechanical SBO. She was subsequently taken to the operating room for emergent diagnostic laparoscopy, which showed pan dilatation of small and large bowels without adhesions, hernia, or masses, suggesting severe ileus, as can be seen in Figure 3. Post-procedure, the patient remained on a ventilator for 24 hours to avoid aspiration. Due to hypotension, sepsis was ruled out. Infectious workup, including blood cultures and urine culture, was negative. Given the history of autoimmune disorder, a comprehensive rheumatological workup was obtained, and the results were unremarkable, as can be seen in Table 1.

**Table 1:** Laboratory values showing the autoimmune workup performed during the hospitalization. ANA = antinuclear antibody; SLE = systemic lupus erythematosus; TSH = thyroid-stimulating hormone

	Laboratory test	Patient’s value	Normal reference range
Test for hypo/hyperthyroidism	TSH	6.22 µIU/mL	0.45-4.50 µIU/mL
Test to rule out Addison’s disease	Random cortisol	99.3 µg/dL	2.5-19.5 µg/dL
Performed to rule out autoimmune vasculitis	Myeloperoxidase	<0.2 AI	<0.9
Performed to rule out autoimmune vasculitis	Proteinase-3	<0.2 AI	<0.9
To measure antibody levels	IgG	325 mg/dL	694-1618 mg/dL
	IgA	126 mg/dL	81-463 mg/dL
	IgM	<5 mg/dL	48-271 mg/dL
Test to diagnose autoimmune disorders particularly lupus and rheumatoid arthritis	C3 complement	148 mg/dL	90-180 mg/dL
	C4 complement	38 mg/dL	16-47 mg/dL
	Total complement	49 U/mL	31-60 U/mL
Screening test for autoimmune disorders	ANA screen	Negative	Negative
Test to diagnose autoimmune vasculitis	Sm antibodies	Negative	
	Sm/RNP antibodies	Negative	
	Anti SS-B antibody	Negative	
	Chromatin antibody	Negative	
Test to diagnose SLE	DsDNA antibody index	<1.0 IU/mL	<4 IU/mL
	DsDNA antibody	Negative	
	Beta-2 glycoprotein IgG	<1.4 U/mM	<19.9 U/mL
	Beta-2 glycoprotein IgM	<1.5 U/mL	<19.9 U/mL
	Beta-2 glycoprotein IgA	3.1 U/mL	<19.9 U/mL
Test to diagnose antiphospholipid antibody syndrome	Cardiolipin IgA antibody	3.6 U/mL	<19.9 U/mL
	Cardiolipin IgG	<1.6 U/mL	<19.9 U/mL
	Cardiolipin IgM	<1.5 U/mL	<19.9 U/mL

The patient’s final diagnosis was severe Ileus. She was not noted to be on opioid medication that could cause hypomotility of the bowels. The patient was managed medically with gastric decompression via a nasogastric tube. A total of 4 L of bilious output was drained, and she was placed on bowel rest along with an aggressive per rectal bowel regimen that included a Fleet enema and rectal Dulcolax suppository, with successful return of bowel function. Simultaneously, while managing SBO, our patient developed AKI within two days of admission with associated metabolic derangements due to volume depletion from severe fluid losses through nausea, vomiting, and poor oral intake. The patient’s baseline renal function included a creatinine of 0.68 mg/dL and a glomerular filtration rate (GFR) of 100 mL/minute. The patient’s creatinine worsened from 1.58 mg/dL to 3.44 mg/dL, and GFR from 37 mL/minute to 14 mL/minute, within 48 hours of admission. With a urine output of around 300 mL in 24 hours, she was noted to be oliguric, and urinalysis showed hyaline casts. Urine electrolytes and urine eosinophils were not performed. The patient developed AKI due to acute tubular necrosis from hypotension. She was started on hemodialysis as renal replacement therapy, which had to be continued post-hospitalization. When the patient was able to tolerate oral intake, she was discharged to an acute rehabilitation facility. Semaglutide was discontinued before discharge. Upon the latest follow-up, the patient was able to be weaned off hemodialysis as her renal function and urine output improved. Unfortunately, she passed away four months after this hospitalization due to recurrent ileus.

**Figure 4:**
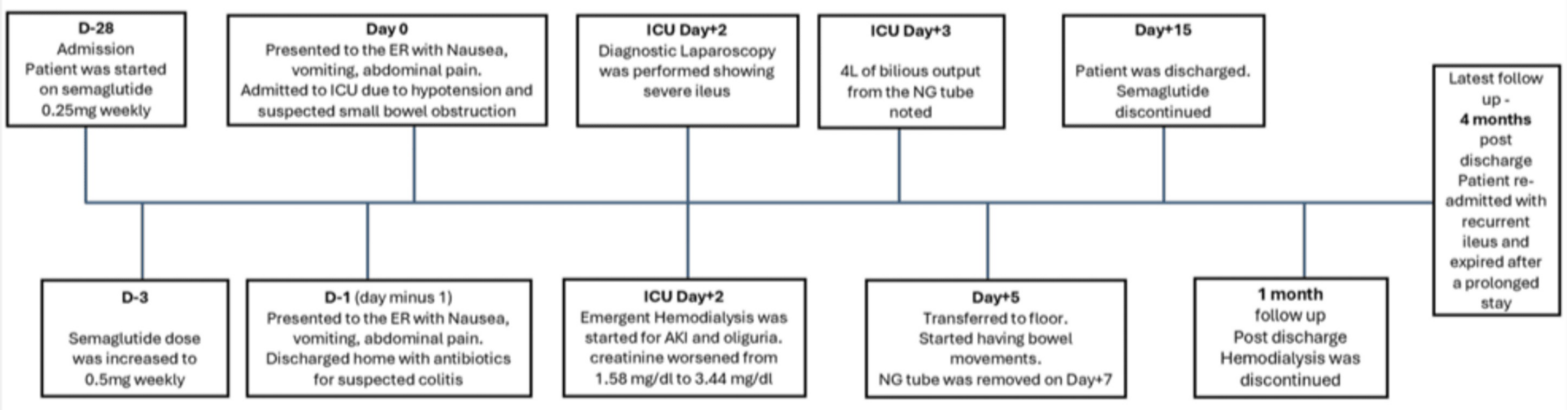
Flowchart illustrating the patient’s clinical course with significant events. AKI = acute kidney injury; ER = emergency room; ICU = intensive care unit; NG = nasogastric

Figure 4 presents the patient’s day-to-day significant events in her clinical course as a flow chart for easier understanding.

## Discussion

Our patient, with increased doses of semaglutide for weight loss and a known history of diverticulitis and granulomatosis polyangiitis, presented with GI complications. Due to the suspicion of mechanical SBO, diagnostic laparoscopy was performed, and she was found to have severe ileus, also referred to as functional SBO. After nasogastric decompression and per rectal enema, her bowel functions returned. However, due to severe fluid loss through the GI tract, the patient developed AKI requiring temporary hemodialysis for several weeks.

It is well known that GLP-1 receptor agonists have GI side effects such as nausea, vomiting, and constipation due to delayed gastric emptying, but bowel obstruction has been rarely reported. On a literature search, there are only a few case reports that mention bowel obstruction with GLP-1 agonists, with most involving diabetic patients.

Interestingly, a few cases of SBO were reported in patients with systemic lupus erythematosus (SLE). Yang et al. [7] reported the case of a 20-year-old female with a history of SLE presenting with recurrent abdominal pain, distension, and weight loss, which was diagnosed as autoimmune enteritic leiomyositis, causing chronic intestinal pseudo-obstruction that was managed conservatively with steroids, antibiotics, nasogastric decompression, and total parenteral nutrition. We performed a battery of rheumatological workup to rule out possible autoimmune etiology of bowel obstruction and AKI in our patient.

Another case report published by Lorenz et al. [8] described a 52-year-old female with no previous surgical history, receiving tirzepatide for weight loss, who was diagnosed with SBO after dose escalation. Her symptoms resolved with conservative management. On the contrary, our patient was directly taken for diagnostic laparoscopy before attempting conservative management. Intraoperatively, she was noted to have severely dilated small and large bowels without inflammation, consistent with profound ileus. From a clinical perspective, our case demonstrates that drug-induced functional bowel disorders can closely mimic mechanical obstruction, potentially resulting in unnecessary invasive interventions if not promptly recognized.

Javed et al. [9] reported the case of a 39-year-old male with diabetes who was started on semaglutide 0.25 mg for four weeks. The dose was increased to 0.5 mg once a week just before the onset of nausea and bilious vomiting accompanied by severe generalized abdominal pain and distension. Investigations revealed elevated fecal calprotectin. Subsequent gastroscopy and colonoscopy ruled out any pathological condition, suggesting that the initially elevated calprotectin level was due to the accompanying ileitis. Like our patient, the symptoms started when the dose was increased to 0.5 mg, and medication reconciliation confirmed the absence of opioid therapy. The Naranjo algorithm was used in calculating the adverse drug reaction probability score. Our patient’s score of 4 suggests a possible temporal association of the symptoms with the medication. Our case highlights the importance of comprehensive medication review in evaluating bowel obstruction and the need for drug safety monitoring.

The pathophysiology of GLP-1 receptor agonist-induced bowel obstruction is multifactorial. GLP-1 receptors are present throughout the GI tract, more significantly in the ileum [10]. Their activation causes vagal nerve stimulation, resulting in delayed gastric emptying, altering the migrating motor complex, which regulates gut motility during fasting periods, leading to gastric distension. GLP-1 receptor activation directly inhibits small intestinal motility. This delayed transit through the stomach and small intestine increases the risk for gastroparesis and bowel obstruction, particularly in individuals with preexisting motility issues [11]. Most importantly, the patient’s death four months later due to recurrent ileus, which is several months after semaglutide discontinuation, challenges a causal interpretation and suggests the possibility of persistent GI dysmotility from prior exposure or an unmasked underlying predisposition. Further randomized controlled trials are needed to identify the motility effects or structural changes caused by GLP-1 agonists on the GI system.

Our patient developed AKI with oliguria progressing to anuria, requiring temporary dialysis for a few weeks. Current evidence suggests that GLP-1 receptor agonists do not increase the overall risk of AKI at a population level [12]. Volume depletion due to GI side effects, causing nausea, vomiting, and other intrinsic mechanisms, may contribute to renal dysfunction. There are some cases of AKI with GLP-1 receptor agonists due to acute interstitial nephritis, with full recovery of renal function without usually requiring dialysis [13]. Studies even suggest that semaglutide may reduce the progression of nephropathy in patients with diabetes and chronic kidney disease, as reported in the semaglutide once weekly (FLOW) trial [14].

## Conclusions

This case suggests a potential association between semaglutide dose escalation and functional SBO complicated by AKI. This case emphasizes reviewing medications and keeping GLP-1 receptor agonists as a differential diagnosis in the etiology of bowel obstruction and considering conservative management before proceeding with invasive testing. Clinicians should educate patients about warning signs, monitor for complications, and intervene promptly when severe GI or renal symptoms arise while on semaglutide.
